# Process evaluation of a complex workplace intervention to prevent musculoskeletal pain in nursing staff: results from INTEVAL_Spain

**DOI:** 10.1186/s12912-021-00716-x

**Published:** 2021-10-06

**Authors:** Mercè Soler-Font, José Maria Ramada, Antoni Merelles, Anna Amat, Carmen de la Flor, Olga Martínez, Claudia Palma-Vasquez, Consuelo Sancho, Pilar Peña, Ute Bültmann, Sander K. R. van Zon, Consol Serra

**Affiliations:** 1 Center for Research in Occupational Health, University Pompeu Fabra/ IMIM (Hospital del Mar Medical Research Institute), Barcelona, Spain; 2 CIBER of Epidemiology and Public Health, Madrid, Spain; 3 Occupational Health Service, Parc de Salut Mar, Barcelona, Spain; 4 Nursing Department, Nursing and Podiatry Faculty, University of Valencia, Valencia, Spain; 5 Occupational Health Service, Corporació Sanitària Parc Taulí, Sabadell, Spain; 6 Department of Health Sciences, Community and Occupational Medicine, University of Groningen, University Medical Center Groningen, Groningen, The Netherlands

**Keywords:** Participatory, Ergonomics, Health promotion, Mindfulness, Mediterranean diet, Nordic walking, Case management, Cluster randomized controlled trial

## Abstract

**Background:**

INTEVAL_Spain was a complex workplace intervention to prevent and manage musculoskeletal pain among nursing staff. Process evaluations can be especially useful for complex and multifaceted interventions through identifying the success or failure factors of an intervention to improve the intervention implementation.

**Objectives:**

This study performed a process evaluation of INTEVAL_Spain and aimed to examine whether the intervention was conducted according to the protocol, to investigate the fulfilment of expectations and the satisfaction of workers.

**Methods:**

The intervention was a two-armed cluster randomized controlled trial and lasted 1 year. The process evaluation included quantitative and qualitative methods. Quantitative methods were used to address the indicators of Steckler and Linnan’s framework. Data on recruitment was collected through a baseline questionnaire for the intervention and the control group. Reach and dose received were collected through participation sheets, dose delivered and fidelity through internal registries, and fulfilment of expectations and satisfaction were collected with two questions at 12-months follow-up. Qualitative methods were used for a content analysis of discussion groups at the end of the intervention led by an external moderator to explore satisfaction and recommendations. The general communication and activities were discussed, and final recommendations were agreed on. Data were synthesized and results were reported thematically.

**Results:**

The study was performed in two Spanish hospitals during 2016-2017 and 257 workers participated. Recruitment was 62 and 51% for the intervention and the control group, respectively. The reach of the activities ranged from 96% for participatory ergonomics to 5% for healthy diet. The number of sessions offered ranged from 60 sessions for Nordic walking to one session for healthy diet. Fidelity of workers ranged from 100% for healthy diet and 79% for participatory ergonomics, to 42 and 39% for Nordic walking and case management, respectively. Lowest fidelity of providers was 75% for case management and 82% for Nordic walking. Fulfilment of expectations and satisfaction ranged from 6.6/10 and 7.6/10, respectively, for case management to 10/10 together for the healthy diet session. Discussion groups revealed several limitations for most of the activities, mainly focused on a lack of communication between the Champion (coordinator) and the workers.

**Conclusions:**

This process evaluation showed that the implementation of INTEVAL_Spain was predominantly carried out as intended. Process indicators differed depending on the activity. Several recommendations to improve the intervention implementation process are proposed.

**Trial registration:**

ISRCTN15780649.

**Supplementary Information:**

The online version contains supplementary material available at 10.1186/s12912-021-00716-x.

## Background

Musculoskeletal pain (MSP) affects people across their life-course in all regions of the world [1]. In Europe, musculoskeletal conditions represent 60% of permanent disabilities and 50% of sickness absences [2]. The nursing staff (i.e., nurses and nursing aides) is an occupational group at high risk of MSP due to heavy manual lifting to mobilize patients [3–5]. Several European studies found that between 70 and 80% of nurses reported MSP [4, 6]. Moreover, nurses and nursing aides with daily patient-handling had twice the risk of developing work-related back injuries compared with nurses without daily patient-handling [7, 8].

MSP is influenced by a complex and dynamic interaction between biological, psychological and social factors [9–11]. Engel (1977) proposed the biopsychosocial model for a better understanding of health and illness. The biopsychosocial model is the dominant framework in the field of MSP, which considers that pain and a person’s capacity to manage it are modulated by the interaction of biological, psychological and social factors [11]. In the workplace, MSP could include biological factors (i.e., age, sex, genetics), psychological and cultural factors (i.e. health beliefs, expectations, fear, somatization tendency), lifestyle factors (i.e. physical activity, diet, toxic habits as smoking and alcohol abuse), the work-related environment (i.e. tasks, equipment/tools, and organization), organization safety culture and social context (i.e. labour market, social security systems, national health systems) [5, 8, 10, 12–14]. Several workplace interventions have been implemented in different working populations, including nursing staff, to prevent and reduce MSP and to promote an early return to work after sickness absence, and multifaceted interventions have shown to be more effective than those based on a single component [12–20].

To prevent and manage MSP in nursing staff in the workplace, a multifaceted intervention encompassing three prevention levels (INTEVAL_Spain) was developed [21]. Components of the intervention were participatory ergonomics (primary prevention); healthy lifestyle promotion programme with Nordic walking, mindfulness, and healthy diet session (primary prevention); and tailored case management (secondary and tertiary prevention). The intervention effectiveness was evaluated in an intention-to-treat cluster randomized controlled trial with the primary outcomes of reduction of MSP and sickness absence and lasted 1 year [21, 22]. At 1 year follow-up, the effect evaluation showed that the intervention group had a significantly lower prevalence of MSP at the neck, shoulders and upper back compared to the control group [22]. No effects were found regarding the reduction of sickness absence [22].

This study concerns a structured process evaluation of the INTEVAL_Spain intervention. A process evaluation is fundamental to understand the factors for success or failure of an intervention [23, 24]. Process evaluations can be especially useful for complex and multifaceted interventions, because the identification of the success or failure factors of an intervention can be used also as a feedback tool to improve the intervention itself, help to interpret the outcomes, and contribute to the generalizability, applicability and transferability of intervention studies [25]. Therefore, we aimed to develop a process evaluation to improve implementation process through examining whether INTEVAL_Spain was conducted according to the protocol (i.e., the intervention was implemented as intended), and investigating the fulfilment of expectations and general satisfaction of nursing staff who participated in the intervention. The Steckler and Linnan’s framework was used to develop, plan and guide this process evaluation. It included seven indicators: recruitment, reach, dose delivered, dose received, fidelity, fulfilment of expectations and satisfaction [25–27].

## Methods

### Study design

The process evaluation was part of INTEVAL_Spain, a two-armed cluster randomized controlled trial with an intention-to-treat intervention. Detailed information on the content, methodology and evaluation of INTEVAL_Spain has been described previously [21, 22, 28]. Participation was voluntary and signed informed consent was obtained from all workers who agreed to participate in the study. Ethical clearance was granted to the authors prior to the study by the Clinical Research Ethical Committee of Parc de Salut Mar (reference number: 2014/5714/1).

### Context

In Spain, hospitals usually have an in-house occupational health service (OHS). Their tasks include health surveillance mainly through health examinations, risk assessment, investigation of occupational injuries, job adjustments, workplace adaptations, training and giving information of both, occupational and non-occupational health risks. INTEVAL_Spain was conducted from September 2016 to December 2017 in two tertiary hospitals in the Barcelona province (Barcelona city and Sabadell) with an experienced in-house OHS, and a workforce of 4000 workers each, of whom around 60% are nursing staff.

### Study population

Clusters were eight independent hospital units, and each cluster was had between 20 to 60 nurses and nursing aides, who worked in the morning, afternoon or night shifts. According to the annual OHS risk assessments, the selected clusters were exposed to high physical demands (e.g., prolonged standing, patient-handling mobilizations). The complete nursing staff from these units was eligible for the study, including those on sickness absence. Exclusion criteria were temporary nursing staff who were on sabbatical leave, who worked in several units and who had worked for short periods of time (less than 3 months).

### Procedure

The procedures used to recruit the nursing staff consisted mainly of informative sessions performed at the work units before randomization. These sessions were moderated by the head of the OHS, the project Champion (study coordinator) and the ward supervisors and had a duration of approximately 45 min. The structure of the informative sessions was 1) presentation of the study, 2) explanation of the intervention, and 3) delivery of the informed consents and the baseline questionnaires. Likewise, additional informative sessions were held in the intervention clusters before starting each activity of the intervention (i.e., participatory ergonomics, Nordic walking, Mindfulness, healthy diet and case management).

### Intervention

The intervention consisted of three main components that included five activities: participatory ergonomics, a healthy lifestyle promotion programme including Nordic walking, mindfulness and healthy diet activities, and a tailored case management programme that included several referral services. Table 1 describes the intervention components and activities. Moreover, both, the intervention and the control group received the usual care of OHS, as described elsewhere [21, 22].

**Table 1:** Intervention components and activities: descriptions and planned doses

Component	Activities	Description	Dose
**Participatory ergonomics**	–	The ERGO group consisted of the ergonomist, the project champion, a volunteer nurse and nursing aide from each shift (morning, afternoon and two-night shifts), the unit supervisor/s, and one prevention delegate (union representative). They met three times. In the first meeting, the results of the unit questionnaire were presented, and an ergonomics training was carried out. In the second meeting, ergonomic problems in the unit were identified and prioritized. In the third meeting, the proposal of preventive measures and a final report were developed. In between these meetings, the volunteers of the ERGO group involved their co-workers to provide input for the meetings.	The ERGO group held an in-door weekly meeting of one hour each for three weeks.
**Healthy lifestyle promotion programme**	Nordic walking	Nordic walking training (outdoor) carried out by an expert trainer.	A 12 sessions weekly programme of 1.5 h/session.
	Mindfulness	Indoor course based on Mindfulness-based Stress Reduction (MBRS) developed by an expert psychologist.	Four sessions of 2 h/session per week.
	Healthy diet chef session	An indoor-formative session carried out by an expert chef.	One session of 3 h.
*Tailored case management programme*	Motivational follow up	Phone service carried out by the case manager to make sure that the nurse and/or nursing aide was doing the planned service sessions, to monitor his/her emotional and physical status, and to provide positive reinforcement.	Phone call every two weeks.
	Education of Health beliefs	A physiotherapist or an occupational health nurse led a session focused on chronic pain, myths related to pain, understanding the role of drugs, physical activity, and stress in managing MSP. The session started and finished answering the Fear Avoidance Beliefs Questionnaire (FABQ) [29]. During the session the patient watched a chronic pain video^a,b^ and received a leaflet with all the information of the session.	One session of 45 min.
	OHS physician	An Occupational Health physician consulted nursing staff when their pain or discomfort was not diagnosed previously, to discard red flags and referred to rehabilitation if appropriate.	One medical consultation of 45 min, and a follow-up of 30 min if the physician considered that was necessary.
	Rehabilitation	Rehabilitation consisted of a consultation with the rehabilitation physician and a physiotherapy rehabilitation programme.	One first consultation of 12,5 min with the rehabilitation physician, and a daily physiotherapy programme of 50 min sessions (with the possibility of three days face-to-face and two days exercises from home). The physiotherapy programme duration was adapted to each worker.
	Cognitive-behavioural therapy (CBT)	CBT focused on stress management at work developed by an expert psychologist.	Six sessions of one hour each, every two weeks. The number of sessions could be expanded if needed.

^a^Health Hunter New England Local Health Discrtirct. NSW Government. Brainman: understanding pain and what to do about it. [internet video]. HNE health. 2016 [accessed 9 Feb 2021 Available in: https://youtu.be/qEWc2XtaNwg

^b^Sociedad Española del dolor SED. Vídeo explicadivo sobre dolor crónico [internet video]. Youtube. 2014 [accessed 9 Feb 2021]. Available in: https://www.youtube.com/watch?v=JYA_mrNuLz0

All components of the intervention were coordinated by a project Champion who organized and led the field work (i.e., planning the activities calendar, informative sessions, research team meetings, collecting and processing data, and writing reports, promoting the participation of workers in the study). The project Champion was an expert in charge to perform these tasks along the study.

Moreover, each component was carried out by expert providers. The participatory ergonomics provider was an ergonomist; Nordic walking and mindfulness training was implemented by known expert instructors and for healthy diet was a chef. Case management was implemented by a trained case manager for the motivational follow-up, and the occupational physician, physiotherapist, rehabilitation physician and physiologist for the referral services.

*Participatory ergonomics* was led by an expert ergonomist of the OHS of each corresponding hospital. This component was based on the ERGOPAR Method [30] which was tested previously in Spanish companies with positive results [31, 32]. The ERGOPAR Method consisted of three phases: diagnostics, treatment and implementation. The diagnostic phase consisted of administering a validated self-completed questionnaire about MSP and exposure to musculoskeletal risk factors at work. The treatment phase consisted of the implementation of participatory ergonomics itself by identifying problems, proposing solutions and prioritizing them (see Table 1). The implementation phase consisted of the execution of preventive measures that included technical, structural, and organizational improvements in the workplace and training/information. The head of the OHS coordinated an “Operative group” including the key managers of the hospital for the implementation of the measures (e.g., managers from the Human Resources Department, Economics Department, Maintenance/Cleaning Department, etc.) with monthly meetings to follow-up the implementation process using a standardized planning table.

The *healthy lifestyle promotion programme* included activities of Nordic walking, mindfulness and healthy diet based on the Mediterranean diet. All these activities were led by experts of each area. More detailed information has been described previously [21].

*The tailored case management programme* for nursing staff with some work limitations due to MSP consisted of the early detection of disabling musculoskeletal conditions (MSP and/or musculoskeletal disorders) and support of return to work, through a multidisciplinary and priority care system. Workers were voluntarily referred to case management either by proposal of a physician of the OHS, by the unit supervisor or on their own initiative. A trained case manager performed a telephone interview using a questionnaire constructed of validated instruments (i.e. Start Back Screening Tool [33, 34], 12-Item General Health Questionnaire [35], European Quality of Life Five Dimension Three Level Scale Questionnaire [36], Generalized Self-Efficacy scale [29], Fear Avoidance Beliefs Questionnaire [37], Self-reported multimorbidity list [38], and a validated question of somatization [8]) to generate a risk profile and to assign workers to three strata of management and treatment, according to their level of risk for persistent musculoskeletal symptoms: low, medium or high. Nurses and nursing aides assigned to the low-risk group attended an education session on health beliefs related to MSP. The ones assigned to the medium and high-risk groups attended also the education session on health beliefs. Besides, medium and high-risk cases were discussed at the weekly clinical session with members of the OHS to decide the specific treatment including rehabilitation, medical consultation with an OHS physician, and cognitive-behavioural therapy, and possible specific needs at work such as job adjustments or workplace adaptations. The algorithm of Case Management is shown in Supplementary Fig. 1.

### Process evaluation

The process evaluation of INTEVAL_Spain included both quantitative and qualitative methods.

### Quantitative methods

Quantitative methods were used to address the indicators of Steckler and Linnan’s framework for process evaluations of public health interventions [26]. Recruitment was measured for both the intervention and control group. Further process indicators for the intervention group were reach, dose delivered, dose received, fidelity, fulfilment of expectations and satisfaction.

Process evaluation data were collected on worker level (i.e., actions pertaining to the workers) and on provider level (i.e. actions pertaining to the provider). Recruitment, reach, dose received, fulfilment of expectations and satisfaction were collected at worker level. Dose delivered was collected on provider level. Fidelity was collected on both worker and provider level. The main reasons for non-participation were collected at the 12-month follow-up questionnaire. All indicators of the process evaluation, their definition and data collection methods are described in Table 2. The process indicator data were analysed by means of descriptive statistics (mean, standard deviation, median, percentage).

**Table 2:** Process evaluation indicators, definitions and data collection

Indicator	Level		Data collection
Recruitment	Workers	Number of nursing staff who answered the baseline questionnaire of all nursing staff assessed for eligibility that met the inclusion criteria. Based on ERGOPAR Method recommendations we expected a 60% recruitment.	Signed consent and baseline questionnaires.
	Provider	NA	
Reach	Workers	Number of nursing staff who attended at least 75% of the sessions of the corresponding activity. The research team established a threshold of 75% of minimum attendance of each activity to consider the activity reached.	Participation sheet in each session collected by each expert^a^
	Provider	NA	
Dose delivered	Workers	NA	Champion internal registry
	Provider	The total sessions offered throughout the intervention, and for each component thereof.	
Dose received	Workers	Defined as the extent to which nursing staff actively participated in each component of the intervention. The variable was operationalized as the number of sessions that the nursing staff attended in each activity. This variable was categorized into three categories (1) < 50% of the sessions, (2) 50-74%, and (3) 75-100%.	Participation sheet in each session collected by each expert^a^
	Provider	NA	
Fidelity	Workers	Number of nursing staff who attended > 75% of sessions from those who were enrolled to participate.	Champion internal registry
	Provider	The number of sessions performed from those planned according to the protocol. When this item was performed according to the protocol, a fidelity score of 100% was reached. The expected fidelity at provider level for the development of the activities was at least 85% due to that expert professionals were contracted to carry out the activities, and the number of sessions to develop was agreed with them^b^.	
Fulfilment of expectations	Workers	Assessed through the following question asked to the nursing staff: “Did this activity/course meet your expectations?” The answers were given on a Likert scale ranging from 1 to 10, with 10 indicating the highest fulfilment of expectations.	A fulfilment of expectations question at the end of each component.
	Provider	NA	
Satisfaction	Workers	Assessed for all the components of the intervention through the following question asked to the nursing staff: “In general, what is your satisfaction with the activity/course?”. The answers were given on a Likert scale ranging from 1 to 10, with 10 indicating the highest level of satisfaction.	A satisfaction question at the end of each component.
	Provider	NA	

*NA* Not available.

^a^Experts were the Ergonomist, Nordic Walking trainer, mindfulness instructor, Chef and case manager

^b^Number of sessions of each activity are specified in Table 1

The analyses were performed with STATA 16 (StataCorp, 2016, Stata Statistical Software: release 16. College Station, TX: StataCorp LP).

### Qualitative methods

Additionally, qualitative methods were used to analyse the 2-h discussion groups. A total of three independent discussion groups with stakeholders were held at the end of the intervention to discuss the satisfaction and to propose recommendations for the implementation process. Two groups were comprised by the nursing staff of the hospitals (i.e., one group for each hospital). The nursing staff that participated in the discussion groups were the volunteers that previously constituted the ERGO group for the participatory ergonomics and included a worker from each shift (morning, afternoon and two-night shifts), the supervisors of the unit, and the prevention delegate (union representative) of each hospital unit. The last group was comprised by the professionals who led the implementation of the intervention (i.e., the head of OHS of both hospitals and the champion).

The discussion groups were led by an external moderator. The moderator had a script with the themes to discuss. There were two main themes: general communication (quality and media) and the activities. Each activity was discussed in the categories of format (duration of the activities and the sessions) and quality (place, material and providers). During the discussion the moderator introduced the themes and were discussed. After, to close the discussion groups, several recommendations to improve the implementation process were agreed. These data were collected and reported in a document sheet during the session. Data were extracted and organized to proceed with the content analysis within the discussion groups. Finally, categories were synthesized, and results were reported thematically [39].

### Deviations from study protocol

There was a deviation from the protocol regarding the context indicator. In the protocol we planned to collect its indicator through the discussion groups, and through 6 and 12-month follow-up questionnaires with three questions related to aspects related to their usual workload (improvements of the manual handling of patients, technical aids, and load handling). Focusing on the context definition of Steckler and Linnan’s framework “aspects of the larger social, political, and economic environment that may influence intervention implementation” we finally considered more adequate to provide a written description of context, than using three questions proposed in the protocol, as they were focused on the effectiveness of the intervention instead of the process.

## Results

### Recruitment

**Fig. 1:**
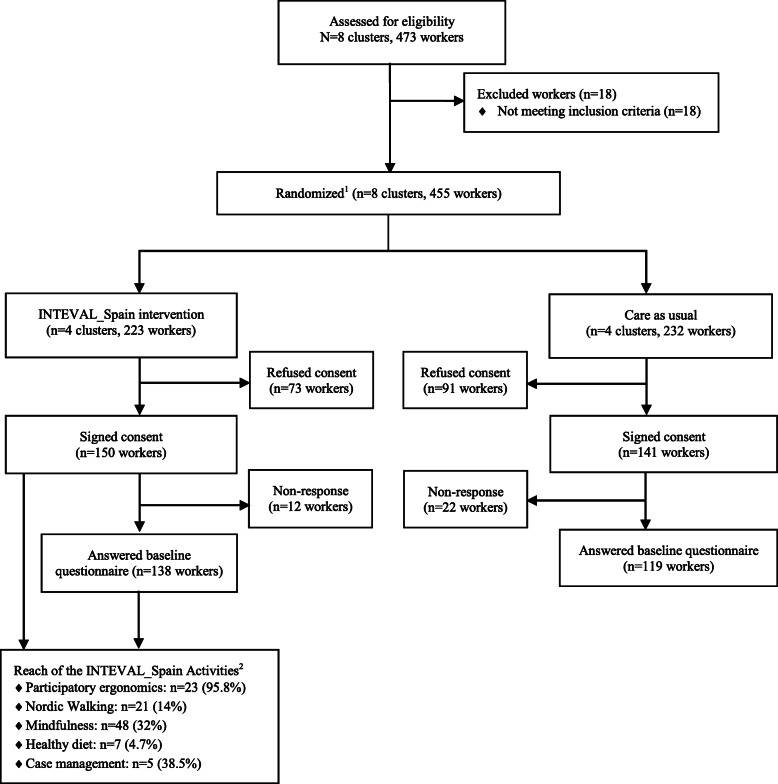
Flowchart of worker recruitment and reach indicators. ^1^Randomization was at cluster level (hospital units were randomized). ^2^Participation was voluntary and free. For participatory ergonomics reach was calculated as the proportion of nursing staff who attended at least the 75% of the sessions from the number of expected participants (i.e., six workers per cluster: a volunteer nursing staff from morning, afternoon and two-night shifts, the unit supervisor/s, and one prevention delegate). For the activities targeted to overall nursing staff (i.e., Nordic walking, mindfulness and healthy diet), reach was calculated as the proportion of nursing staff who participated in these activities and attended at least the 75% of the sessions from those who signed the informed consent. Finally, for case management, reach was calculated as the proportion of nursing staff who participated in this component and attended at least the 75% of the sessions from those who the intervention was recommended (i.e., the sum of nursing staff that participated and the nursing staff that declined the intervention)

At baseline, the study included eight clusters with 473 nursing staff. After the exclusion of 18 nursing staff that did not meet inclusion criteria, the intervention group included 223 and the control group 232 nurses and nursing aides. Of these, 67.3% (*n* = 150) of nursing staff in the intervention group and 60.8% (*n* = 141) of the control group signed the informed consent. As shown in Fig. 1, 138 nursing staff in the intervention group (61.9%) and 119 in the control group (51.3%) completed the baseline questionnaire.

### Reach

Twenty-three nurses and nursing aides (95.8%) attended at least 75% of the participatory ergonomics sessions of the 24 expected. The healthy lifestyle promotion programme was available for all the nursing staff that signed the informed consent (*n =* 150). Of these, a total of 48 nursing staff (32.0%) attended at least 75% of the mindfulness sessions, 21 (14%) in Nordic Walking and 7 (4.7%) in the healthy diet sessions. Finally, case management was targeted to 13 nurses and nursing aides and 5 (38.5%) attended at least 75% of their planned sessions (Fig. 1). The main reasons reported for non-participation in the activities were “difficulties to meet schedules” and “lack of time”.

### Dose delivered

Taken together, activities included a total of 137 sessions offered at provider level. The activity with the highest number of offered sessions was Nordic walking (60 sessions) followed by case management with 48 sessions, mindfulness (16 sessions), participatory ergonomics (12 sessions) and healthy diet session (1 session) (Table 3).

**Table 3:** Results of process evaluation indicators of each component and overall intervention

Process indicators	Total	Participatory Ergonomics	Nordic Walking	Mindfulness	Healthy Diet	Case Management
**Dose delivered**						
N° of sessions (%)	137 (82.0)	12 (100.0)	60 (81.7)	16 (100.0)	1 (100.0)	48 (75.0)
**Dose received**^**a**^						
< 50% [n°workers (%)]	30 (18.1)	3 (10.3)	12 (24.0)	11 (16.4)	0 (0.0)	4 (30.8)
50-74% [n°workers (%)]	32 (19.3)	3 (10.3)	17 (34.0)	8 (11.9)	0 (0.0)	4 (30.8)
≥ 75% [n°workers (%)]	104 (62.7)	23 (79.3)	21 (42.0)	48 (71.6)	7 (100.0)	5 (38.5)
**Satisfaction**						
Expectations [mean (SD)]	8.6 (1.9)	–	8.6 (1.3)	8.8 (1.6)	10 (0.0)	6.6 (2.0)
General [mean (SD)]	8.9 (1.4)	8.8 (1.3)	8.8 (1.0)	9.6 (1.0)	10 (0.0)	7.6 (2.3)

^a^One hundred thirty nursing staff participated in the activities. Of them, 23 participated in two activities, five participated in three activities, and one participated in four activities

### Dose received

One hundred thirty nursing staff participated in the activities, of them, 23 participated in two activities, five participated in three activities, and one participated in four activities. Hence, there were 166 participations in total (Table 3). Of these, 104 nursing staff (62.7%) received more than 75% of the delivered sessions, followed by 32 workers (19.3%) that attended between 50 to 74% of the sessions, and 30 (18.1%) that received less than the 50% of the sessions.

### Fidelity

#### Worker level

In total, 29 nursing staff were inscribed to participate in participatory ergonomics, and of these 23 (79.3%) attended between 75 and 100% of the sessions, followed by 48 nurses and nursing aides of the 67 inscribed in mindfulness (71.6%), and 7 in the healthy diet session (100%). For Nordic walking, 29 nurses and nursing aides of the 50 inscribed (58.0%) and 8 of the 13 inscribed for case management (61.5%) attended less than 75% of the sessions.

#### Provider level

Overall fidelity was 82%. Fidelity for participatory ergonomics, mindfulness and healthy diet session were 100%. For Nordic Walking and case management fidelity was 81.7 and 75%, respectively.

### Fulfilment of expectations

Overall fulfilment of expectations was 8.6/10 (SD: 1.9). For healthy diet it was 10.0/10 (SD:0.0), followed by mindfulness 8.8/10 (SD:1.6) and Nordic walking 8.6/10 (SD:1.3). Case management showed the lowest fulfilment of expectations 6.6/10 (SD:2.0). Fulfilment of expectations was not available for participatory ergonomics.

### Satisfaction with the intervention and the activities

General satisfaction was 8.9/10 (SD: 1.4). Satisfaction ranged from 10.0 /10 (SD:0.0) and 9.6/10 (SD: 1.0) for healthy diet and mindfulness, respectively, to 7.6/10 (SD: 2.3) for case management.

### Discussion groups

Table 4 shows the thematic synthesis of the discussion groups. For general communication, the discussion groups reported that the use of WhatsApp was the most positive means of communication. A lack of communication was also identified as some nursing staff reported not being sufficiently informed about the project and activities. Several limitations were identified: (1) limited communication about the implemented measures of participatory ergonomics; (2) a limited interest in the Nordic Walking activity; (3) limited attendance of the healthy diet session as only one session was offered for all participants; (4) limited communication about what case management consisted of and how to access the case management; and (5) delays in the referral services of case management programme. The main recommendation focused on improving communication in the activities and offering the activities tailored to all shifts.

**Table 4:** Results of the discussion groups by themes: satisfaction and recommendations

Themes	General Satisfaction	Recommendations
**General Communication**	▪ The nursing staff does not use often the job email so the communication through WhatsApp groups was well appreciated (WG1, PG). ▪ There was a limited communication between the champion and the nursing staff, some nursing staff considered not to be enough informed about the project and the activities (PG).	▪ To use of WhatsApp groups as the main communication media. ▪ To perform periodic meetings to keep the nursing staff updated regarding to the activities.
**Participatory ergonomics**	▪ The format, duration and organization ERGO groups were valued positively, but after this, nursing staff sometimes were unaware of the implemented measures (WG1, WG2, PG).	▪ To inform nursing staff each time a measure is implemented.
**Nordic walking**	▪ The format, duration and organization were adequate, but some nursing staff considered that they did not want to spend their “free time” doing this activity (WG1).	▪ To improve knowledge about the advantages of Nordic walking for improving health.
**Mindfulness**	▪ The format, duration and organization were adequate (WG1, WG2, PG).	▪ –
**Healthy diet**	▪ The session with the chef involved traveling to Barcelona, and limited nursing staff to the afternoon and night shifts, but it was very interesting and well valued for the workers (WG2, PG).	▪ To offer at least one session per shift.
**Case management**	▪ Limited communication, the nursing staff did not remember what it consisted of and did not understand it (WG1, PG). ▪ Delays to access to the rehabilitation service as this service required a referral request managed by the OHS (PG).	▪ To reinforce the communication and knowledge of case management to the nursing staff. ▪ To eliminate intermediaries or seek measures to accelerate the process of access to the rehabilitation service.

*WG1* Worker’s discussion group 1(Barcelona), *WG2* Worker’s discussion group 2 (Sabadell), *PG* Professional’s discussion group.

## Discussion

The results showed a good recruitment for the intervention group but a slightly lower than expected recruitment for the control group. Mindfulness indicators of dose delivered, fidelity at provider level, fulfilment of expectations and satisfaction were high; and reach, dose received and fidelity at worker level were moderate. Nordic walking showed high fulfilment of expectations and satisfaction, moderate dose delivered and fidelity at provider level, and low reach, dose received and fidelity at worker level. Healthy diet showed good results according for most indicators except for reach. Case management showed moderate reach and the worst results for the other indicators. Discussion groups found several limitations for most of the activities except for mindfulness.

The recruitment was adequate for the intervention group, but was lower than expected for the control group, although nursing staff did not know the group assignment during recruitment. The inclusion of the surgical unit in the control group, where only 39% of the nursing staff answered the questionnaires, might explain the low recruitment (data not shown). Informative meetings in the surgical unit were challenging as attendance depended on the shifts and several workers were not able to attend. Therefore, the timing of the informative meetings during recruitment should had been more flexible for this unit. Finally, the surgical unit was in an organisational restructuring process during the study and left the trial [22].

Reach differed according to the activities as the number of nursing staff enrolled to participate ranged from seven in the healthy diet session to 67 in the mindfulness sessions. There may be several reasons for the difference in reach. For participatory ergonomics a participation of six nurses and nursing aides per cluster was expected; for the healthy lifestyle promotion programme the participation was open to all of the 150-nursing staff who signed the informed consent; and the tailored case management was targeted only to nurses and nursing aides with MSP (i.e., 13 nurses and nursing aides were targeted). Consequently, to calculate the reach we have considered the number of expected nursing staff in each activity as a denominator. Secondly, the discussion groups identified a lack of communication, as several nursing staff reported not being sufficiently informed about the intervention and activities. In this regard, the reach could be improved by increasing the number of informative meetings before the start of each activity. At these meetings, a clear contact means should also be provided to the nursing staff, for example, the Champion’s phone number and/or email address. These initiatives would be especially useful for the case management programme, where discussion groups identified a lack of understanding what the programme consisted of. Third, reach of the health promotion activities may differ due to the number of sessions offered, e.g., healthy diet consisted of only one session for the intervention group. Fourth, the date and hour of the healthy diet session was agreed upon only with the chef and not with the nursing staff and had the lowest reach. Thus, to increase reach it is recommended that the date and time slots of the activities should be agreed upon with the nursing staff. Moreover, offering at least one session per shift and aligning the calendar of the workers with the healthy diet chef could have been crucial to this activity. This may be especially important as the healthy diet session had the highest satisfaction and the lowest reach, but it might still be an interesting addition to the intervention programme. Finally, it is necessary to involve nursing staff in the decision process, not only to establish the activity calendar, but also to agree which evidence-based activities should be offered within the health promotion programme to fit with their interests [40].

The fidelity of workers enrolled to participate in the activities was usually high. Case management had the lowest fidelity (38.5%) which means that most of the nurses and nursing aides targeted to the case management programme did not complete it entirely. One reason could be the lack of communication, as the discussion groups concluded that some nursing staff did not understand the case management programme. Furthermore, although case management programmes have been shown to be effective to reduce MSP and to improve the return-to-work process [41–43], it is possible that participants dropped out because their expectations were not met [44]. In addition, the sessions on health beliefs, rehabilitation and cognitive-behavioural therapy were not performed at the workplace, i.e., nurses and nursing aides had to attend these sessions during their leisure time, making participation more difficult.

Fidelity at the provider level was high for participatory ergonomics, mindfulness and healthy diet, but low for Nordic walking and case management. Fidelity for Nordic walking was low because one group had to finish the training a few weeks earlier because the weather was too hot for this outdoor activity held at noon. Therefore, environmental factors must be taken into account when timing and implementing activities. For case management, the services were coordinated by the case manager, who was the link between the nursing staff and the case management referral services (i.e., education on health beliefs, medical consultation with OHS physicians, rehabilitation and cognitive-behavioural therapy). For case management referral services, fidelity was lower for the rehabilitation service (data not shown). The rehabilitation service was the only referral service that was not directly coordinated by the case manager, as it required a referral request from the OHS physician to the rehabilitation service. Previous research has shown that communication between medical specialists can be difficult and may hinder the rehabilitation process and the return-to-work [45]. Also, more intermediates in the case management may require more time and slow down the rehabilitation process, which in turn may demotivate the nurses and nursing aides. It is possible that higher fidelity at the provider and worker level on case management (as case management was focused on nursing staff with MSP or sickness absence) could have resulted in less MSP and lower sickness absence [46].

Fulfilment of expectations and satisfaction were high for most of the activities, except for case management. Case management had also the lowest fidelity at provider and worker levels. It is possible that the lack of fidelity at provider level demotivated the nursing staff, decreasing their expectations, and reducing the fidelity at worker level and outcomes [47].

Finally, the discussion groups identified several limitations, except for mindfulness that discussion groups reported that “format, duration and organization were adequate”. For participatory ergonomics it was identified a lack of communication about the implemented measures; for Nordic walking a limited interest on this activity was reported; and for healthy diet there was only one session in Barcelona for all the nursing staff that limited the attendance. For case management, there was two important limitations: lack of communication as the discussion groups reported that “the nursing staff did not remember what it consisted of and did not understand it”, and the delays to access to the rehabilitation referral service. In summary, most limitations were related to a lack of communication between the Champion and the nursing staff. As mentioned before, it is necessary to improve the communication between the Champion and the nursing staff, as well to empower nursing staff through including them in the decision process i.e.to agree on what activities offer, the date and time slots.

### Strengths and limitations

The main strength of this process evaluation is that we used quantitative and qualitative methods addressing different indicators of the intervention implementation. Our study has also limitations. The main limitation is that the questionnaires were anonymous, and we did not create an identifier for the questionnaires, so we could not link the process evaluation indicators to the results of the effectiveness study to explore and explain the influence of the implementation with the overall results of the intervention [23]. This decision was taken to encourage participation and make nursing staff feel comfortable, as the questionnaires were self-administered at the workplace and it included personal questions and questions about the relationship with the co-workers and the supervisors. Moreover, as not all the indicators were available at both levels, workers and providers, we could not compare the results of both levels. Also, we did not collect information about the reasons why several nurses and nursing aides did not complete the case management programme or why the fulfilment of expectations was low.

### Recommendations for practice and research

The process evaluation of INTEVAL_Spain provides important information about the process indicators for the implementation of this intervention to prevent and manage MSP in nursing staff. Flexibility regarding the timing and number of the informative meetings during the recruitment phase is needed to facilitate the attendance and participation of the nursing staff. Further, flexibility in offering courses at different time slots is needed to ensure that workers on all shifts (i.e., morning, afternoon and night) have the opportunity to participate. The involvement and empowerment of the nursing staff in the decision process of activity selection and planning may also be crucial for the successful implementation of the intervention [44]. We further recommend performing all activities and services (e.g., referral services of the case management programme) at the workplace and during work time, as other locations and activities organized during leisure time may be a barrier to attend the activities. The involvement of intermediates, i.e., the need of a referral to use the rehabilitation service, may slow down the implementation process [48]. Thus, a Champion or a coordinator who is directly in contact with both the nursing staff and the services could promote a faster implementation.

## Conclusion

This process evaluation showed that the implementation of a complex workplace intervention for nursing staff in two Spanish hospitals was predominantly carried out as intended. Process indicators differed depending on the activities. Participatory ergonomics and mindfulness were implemented successfully according to the indicators; healthy diet was well implemented but had low reach, and Nordic walking and case management showed a low level of implementation according to the indicators. Being flexible during the recruitment, offering activities in several time slots and tailored to shifts, involving and empowering the nursing staff in the decision process, carrying out the activities and services at the workplace and eliminating intermediaries between the case manager and referral services may be necessary to further improve the effectiveness of INTEVAL_Spain.

## Supplementary Information

**Additional file 1.**

## Data Availability

The data and materials used for analysis and make conclusion are available from the corresponding author on reasonable request.
