# Supplementary material for: Process evaluation of a complex workplace intervention to prevent musculoskeletal pain in nursing staff: results from INTEVAL_Spain
Source: BMC Nurs. 2021 Oct 6;20:189. doi: 10.1186/s12912-021-00716-x (PMC8493695; doi:10.1186/s12912-021-00716-x)
Supplement: Supplementary file 1 — Additional file 1. [file 12912_2021_716_MOESM1_ESM.docx]

**Supplementary Figure 1.** Algorithm of case management

**Inclusion criteria:**

1. Work in an intervention cluster
2. Have a functionally limiting MSD

**CASE MANAGEMENT**

Case manager (specifically trained)

Other causes

*Telephone contact*

**MODERATE/HIGH RISK**

**LOW RISK**

*Telephone contact*

Managers

Nursing staff

*Telephone contact*

*CM contacts with the different professionals*

**Active worker**

**RISK PROFILE**

**Case manager**

Excluded

- Red flags^a^
- No consent

**MSD**

**Occupational health service**

**(Secretary, nursing)**

**Sickness absence**

**MOTIVATIONAL FOLLOW-UP**

**Case manager**

*Evaluation and derivation*

**CLINICAL SESSION^b^**

*Telephone contact: interview and* ***questionnaires***

**EDUCATION IN HEALTH BELIEFS**

**OCCUPATIONAL HEALTH SERVICE PHYSICIAN**

**REHABILITATION SERVICE**

**COGNITIVE BEHAVORIAL THERAPY**

^a^ Red Flags: Presence of warning signs (severe trauma or minor trauma in patients with osteoporosis, constitutional symptoms: fever, weight loss, malaise), pregnancy or puerperium, morbid obesity (BMI ≥ 40), recent surgery (< 3 months), neurodegenerative disease, decompensated psychiatric pathology, decompensated cardiac or respiratory pathology, and osteoporosis with symptomatic vertebral fractures.

^b^ It will be derived to the different services based on identified needs.
